# Effects of Different Levels of Inclusion of Apulo-Calabrese Pig Meat on Microbiological, Physicochemical and Rheological Parameters of Salami during Ripening

**DOI:** 10.3390/ani11113060

**Published:** 2021-10-26

**Authors:** Rosa Luisa Ambrosio, Giorgio Smaldone, Marika Di Paolo, Lucia Vollano, Marina Ceruso, Aniello Anastasio, Raffaele Marrone

**Affiliations:** 1 Unit of Food Hygiene, Department of Veterinary Medicine and Animal Production, University of Naples Federico II, Via F. Delpino 1, 80137 Napoli, Italy; rosaluisa.ambrosio@unina.it (R.L.A.); vollano@unina.it (L.V.); marina.ceruso@unina.it (M.C.); anastasi@unina.it (A.A.); raffaele.marrone@gmail.com (R.M.); 2 Department of Agricultural Sciences, University of Naples Federico II, Via Università 100, 80055 Portici, Italy; giorgio.smaldone@unina.it

**Keywords:** fatty acid profile, autochthonous breed, dry-cured meat products, local production

## Abstract

**Simple Summary:**

Consumers’ interest in local food productions is constantly increasing thanks to their well-known nutritional properties. Several authors have demonstrated the influence of meat from indigenous pigs breeds on chemical, physical, and organoleptic features of meat products such as salami. Among the advantages, its effect on the lipid component is undoubtedly the most discussed as it influences their fatty acid profile. In Italy, the Apulo-Calabrese is an example of a pig breed taken up for the enhancement of local productions. However, it is essential to underline the crucial role of the ripening process for cured products, which preserves their quality and improves their appearance and integrity. This research evaluated how Apulo-Calabrese meat influences physicochemical, rheological, and microbiological profiles of salami during the ripening time. The study found that the partial inclusion of 50 and 75% of Apulo-Calabrese meat to the dough of salami reduced the content of saturated fatty acids and improved the appearance by acting on texture and color. The combined effect of the composition of doughs and the ripening process has led to the production of products with a high-quality nutritional profile. The study provides valuable information to manufacturers on how to exploit local pork breeds, suggesting the best cost-benefit combination for them and consumers.

**Abstract:**

This study focused on the characterization of salami produced with meat from different pig breeds. The aim consisted in evaluating the added value of the inclusion of Apulo-Calabrese meat in the production of salami, which was characterized by production until the end of maturation (1, 30, 60, and 120 days). The experimental design involved three types of salami, two of which were produced by partial inclusion of 50 and 75% of the Italian breed pork meat (S50 and S75, respectively). Physicochemical (pH, a_w_, fatty acid analysis, and malondialdehyde concentration), rheological parameters (texture analyses and color measurement), and bacterial biodiversity were evaluated. Results showed that the partial inclusion of Apulo-Calabrese meat influences the fatty acid profile of final products, which were characterized by a higher percentage of monounsaturated fatty acids compared to traditional salami; however, due to the high content of unsaturated fatty acids, S50 and S75 showed higher values of secondary lipid oxidation up to the 120th day. The linoleic and palmitic acid content significantly affected hardness and brightness. Overall, the ripening process was able to control the microbiological profile and the S50 formulation appeared as a suitable choice that could satisfy consumers for nutritional expectations and sensory profiles.

## 1. Introduction

In recent years, a growing interest in the qualitative parameters of pork meat, and in particular in local breed pig products, has been reported [1,2,3,4]. Consumers demand typical products for their high added value and eating quality. Recent studies [3,5] indicate that indigenous pig breeds are characterized by certain differences in chemical, physical, and organoleptic features of meat compared to hybrid pigs. Local pig breeds have been shown to have higher intramuscular fat content than hybrids [5] and are able to deposit more fat due to their distinct lipogenic capacity [6]. According to Wood et al. [7], the traditional pigs grow slowly and are lighter and fatter than the modern pigs at the time of slaughter. The nutritional and healthy information on fats consumed is contradictory. World Health Organization (WHO) includes limitations not only on the amount of fat but also on the food fatty acids compositions [8]. In particular, the replacement of part of the saturated fatty acids (SFAs) in the diet with monounsaturated fatty acids (MUFAs) led to a decrease in inflammation [9] and a reduction in triglycerides, total cholesterol, low-density lipoprotein (LDL) subclasses, and fractions. This causes an antiatherogenic lipid profile [10] that significantly decreases the risk of hypertension [11]. Although local native pig carcasses contain a higher fat content, this is associated with a better fatty acid composition characterized by a higher monounsaturated fatty acid (MUFAs) [2,3,4,5,7] than modern pigs. These characteristics can lead to increased interest in this type of meat and, consequently, in processed products. In the Calabrian zootechnical context, the last decade has seen renewed interest in the Apulo-Calabrese swine breed, previously known as Nero Calabrese [1,12]. The Apulo-Calabrese is one of the six officially recognized Italian native pig breeds: it is a medium-sized autochthonous breed with black skin and black bristles, straight, robust, and longer in the dorsal region [12]. This breed shows good adaptability to different production systems and environmental conditions [12].

Apulo-Calabrese meat is mainly used for the manufacture of typical meat products as salami, including those with the Protected Denomination of Origin (PDO) ones [1]. However, very few studies have emerged from the literature about typical Calabria salami and most of them focused on technological aspects of production and ripening [13,14]. Ripening plays an important role in preserving the quality of these meat products by improving appearance and integrity due to the quality of meat and microbial enzyme activity [15]. This is considered to be one of the most important stages in salami production which clearly influences the characteristics of final dry fermented pork products. In this context, the reconsideration of the link between breed and typical products can be an important tool to increase the value of farming and avoid its decline. To achieve this goal, it is necessary to identify and quantify those properties that best describe the characteristics of the product.

The aim of this paper is to evaluate the effects of different levels of inclusion of Apulo-Calabrese pig meat on microbiological, physicochemical, and rheological traits of salami over the ripening time. In order to enhance the use of native breeds pigs in meat productions, the nutritional quality of salami was also evaluated.

## 2. Materials and Methods

### 2.1. Salami Production and Sample Collection

The study was conducted on three typologies of salami which differed for doughs composition. Therefore, the production was performed using two distinct specimens of pork (*Sus domesticus*), beginning from cuts of commercial crossbreed [Duroc × (Landrace × Large White)] and Apulo-Calabrese pork meat. The three meat doughs were prepared using a defined ratio of raw minced meat: (i) SR salami was obtained from commercial hybrid raw material; (ii) S50 and (iii) S75 salami were made with a percentage of 50 and 75 of Apulo-Calabrese meat, respectively.

Salami were prepared according to a typical local recipe at a local manufacturer. The meat formulations consisted of the shoulder (40% *w*/*w*), ham (40% *w*/*w*), and belly (20% *w*/*w*), both of commercial hybrids and of Apulo-Calabrese breed. The cuts, which were previously unnerved and cut into strips, were minced into a meat grinder, and then divided into three experimental batches of 10 kg (SR, S50, and S75). The mixture spices consisted of salt (250 g), black pepper, both as ground (10 g) and in grains (5 g), 0.15 g/kg potassium nitrate (E252), sausage premix (160 g) containing dextrose, sucrose, sodium ascorbate (E301) and no starter cultures. The paste was stuffed into the natural casing (filzetta), manually tied, hung, and placed in a conditioning chamber. The ripening program was as follows: for the first 12 h a temperature of 22 ± 2 °C without relative humidity (R.H.) and ventilation; within four days, the temperature was reduced to 19 ± 2 °C and R.H. and ventilation were adjusted to 50–60% and 30–80 V/h, respectively; during the last weeks of drying, the temperature was decreased to 12 ± 2 °C, while the R.H. was increased to 75–80%. Whole ripening was carried out for 120 days. For each type of salami (SR, S50, and S75), a total of twelve samples (each weighing about 230 g) were divided into 4 groups, each dedicated to the analysis of a sampling time. Therefore, on day 1 (T0), 30 days (T1), 60 days (T2) and 120 days (T3) 3 samples by type of salami were analyzed.

### 2.2. pH and a_w_ Measurements

The pH measurements were carried out using a digital pH meter (Crison-Micro TT 2022, Crison Instruments, Barcelona, Spain). Water activity (a_w_) was measured by means of Aqualab 4 TE (Decagon Devices Inc., Pullman, WA, USA).

### 2.3. Microbiological Analyses

Ten grams of each sample (casings and salami at different stages of ripening) and 90 mL (1:10, *w*/*v*) of sterilized Peptone Water (PW, Oxoid, Madrid, Spain) were placed in a sterile stomacher bag and homogenized for three minutes at 230 rpm using a peristaltic homogenizer (BagMixer^®^400 P, Interscience, Saint Nom, France). Ten-fold serial dilutions of each homogenate were plated on the surface of appropriate media in Petri dishes. The viable counts of the following micro-organisms were carried out using procedures validated by the UNI CEI EN ISO/IEC 17025 European Standard: (i) total aerobic bacterial counts (TAB) on Plate Count Agar (PCA; Oxoid, Madrid, Spain) incubated at 30 °C for 48/72 h, according to ISO 4833-1:2013; (ii) Total Coliforms on Violet Red Bile Lactose Agar (VRBL, Oxoid, Madrid, Spain), according to ISO 4831:2006; (iii) Lactic Acid Bacteria (LAB) on MRS agar with Tween 80 (Oxoid, Madrid, Spain), incubated at 30 °C for 72 h, according to ISO 15214:2015; (iv) Pseudomonas spp. on Pseudomonas Agar Base with CFC supplement (Oxoid, Madrid, Spain) incubated at 25 °C for 48 h, according to ISO 13720:2010; (v) yeasts and moulds on Dichloran Rose-Bengal Chloramphenicol Agar (DRBC, Oxoid, Madrid, Spain) incubated at 25 °C for 120/168 h, according to ISO 21527:2008. After counting, data were expressed as logarithms of the number of colony-forming units (CFU/g) and means and standard error were calculated.

### 2.4. Fatty Acids Analysis

The extraction of total lipids was performed according to Hara and Radin [16]. A cold transmethylation was performed on fat in agreement with Aboagye et al. [17] with some modification. For the preparation of fatty acid methyl esters about 150 mg of lipid was weighed in a conical vial and dissolved in 3 mL of n-hexane and the mixture was vigorously shaken for 30 s. An aliquot (1.5 mL) of the solution was transferred in a vial and 250 μL of methanolic KOH (c = 2 mol/L) were added. The mixture was vigorously shaken for 30 s three times. 1 μL of the supernatant was analyzed by gas-chromatography (DANI INSTRUMENTS GC 1000) with FID detector, capillary column CP-Select CB for FAME (length 100 m, internal diameter 0.25 mm, film thickness 0.2 μm) and split/splitless injector. The injector and detector temperatures were set at 250 °C. Helium was used as carrier gas at the flow of 1.2 mL/min. The temperature program of the column was 140 °C for 5 min and subsequent increase at 4 °C/min up to 240 °C where it was held for 18 min. Fatty acid methyl esters were identified by comparison of the retention times of the peaks in the sample with previously run pure standard compounds (Supelco^®^ 37 Component FAME Mix, 47885U-Supelco, Sigma-Aldrich, St. Louis, MO, USA). Results are expressed as a percentage of the total fatty acids analyzed and saturated (SFAs), monounsaturated (MUFAs) and polyunsaturated (PUFAs) fatty acids were determined. To establish a nutritional evaluation of salami fat, it was compared three different ripening times (30, 60, and 120 days) from the start of sale. The PUFA/SFA ratio (P/S), the MUFA/SFA ratio (M/S) were determined, and atherogenic and thrombogenic indexes were calculated according to Ulbricht and Southgate [18] and hypocholesterolemic/hypercholesterolemic (h/H) according to Santos-Silva et al. [19] as follows:

Atherogenic Index (AI) = (C12:0 + 4 × C14:0 + C16:0)/[(ΣMUFA + ΣPUFA (n − 6) and (n − 3)](1)

Thrombogenic Index (TI) = (C14:0 + C16:0 + C18:0)/[(0.5 × ΣMUFA + 0.5 × PUFA (n − 6) + (n − 3)/(n − 6)](2)

hypocholesterolemic/Hypercholesterolemic (h/H) = (ΣMUFA + ΣPUFA)/(C14:0 + C16:0)(3)

### 2.5. Rheological Analysis

Texture profile and color measurements have been analyzed from the 30th day (starting time of sale) in order to compare three different ripening times (30, 60, and 120 days), according to Smaldone et al. [20].

#### 2.5.1. Texture Profile Analysis

Texture profile analysis (TPA) was performed according to Marrone et al. [21] with an EZ-Test texturometer Shimaduz (Shimadzu Corporation, Kyoto, Japan) measuring the compression force (Newtons) develop. A cylindrical 25 mm diameter probe of ebonite was used for all TPA tests in this study. Each sample was placed under the probe that moved downwards at a constant speed of 50 mm s-1. The penetration points tested were five: in the center and on four different areas of the surface. The following parameters were evaluated from the TPA curve: hardness (maximum force applied), cohesiveness (strength of internal bonds measured by the ratio between two consecutive compressions), springiness (rate of return after compression), gumminess (hardness × cohesion) chewiness (gumminess x elasticity), resilience (similar to elasticity but expressed as a ratio of energies), adhesiveness (work needed to overcome the forces between the sample and the probe). Each measurement was assessed 7–10 times and the average values were used for statistical analysis.

#### 2.5.2. Color

The color was measured using a Konica Minolta CR 300 colorimeter (Minolta, Osaka, Japan) and color coordinates were measured [21]: lightness (L*), redness (a*) and yellowness (b*), Hue angle (Tan−1 b*/a*), and Chroma [(a*^2 + b*^2) ^ 1/2]. In each instrumental test, five replicates were performed for each sample.

### 2.6. TBARS Analysis

Lipid oxidation was monitored by determining the thiobarbituric acid (C₄H₄N₂O₂S) substances expressed as malondialdehyde (CH₂(CHO)₂) concentration (mg/kg), which represent secondary oxidation products. Measurements were performed according to the method proposed by Settanni et al. [22].

### 2.7. Statistical Analyses

Statistical analyses were performed using SPSS version 26 (IBM Analytics, Armonk, NY, USA). All data were presented as the least square mean (M) ± standard error (sE). The results were analyzed with generalized linear mixed models (GLMMs) and the means were compared using the Tukey test (*p* < 0.05). These models were used to assess whether the inclusion of Apulo-Calabrese meat in salami production influenced the profile of the products. Microbiological, rheological, and physicochemical data were included in GLMMs as dependent variables and the type of salami (SR, S50, S75) was included as the independent variable. Moreover, Pearson’s correlations were calculated between and among microbiological and physicochemical parameters. Statistical significance was predetermined at *p* < 0.05.

## 3. Results and Discussion

### 3.1. Microbiological Analyses

Microbial counts of casings and salami during the ripening period are displayed in Table 1.

Other authors have already deepened the bacteria biodiversity of natural and synthetic casings [23] and highlighted their role in salami microbiological communities. In agreement with Comi et al. [24] and Pisacane et al. [23], in our study, casings were characterized by a Total Aerobic Bacteria at 30 °C of about 5.62 Log (CFU/g). However, if mesophilic bacteria and LAB [23] appear to overlap literature data, the presence of Total Coliforms did not reflect what was reported by other authors especially because of the high contamination (Table 1). For this reason, issues regarding the hygienic state of the casings could be hypothesized.

Regarding microbiological measurements carried out on salami during ripening stages, growth trends of each bacterial population were approximately superimposable among products, showing no significant differences. The mesophilic total bacteria count at the beginning of the trial was high in all samples, ranged between 4.96 and 5.78 Log (CFU/g), and increased until the sixteenth day of fermentation. Afterwards, the TBA count remained stable throughout the last ripening period according to Madonia et al. [2] and to Pisacane et al. [23]. However, S75s made an exception because they were characterized by a gradual TBA increase during the whole ripening period. In addition, our data showed a close relationship between TBA and LAB counts, whose trend defined them as predominant populations. In addition, despite the absence of starter cultures, the high level of lactic acid bacteria could suggest the positive burden of casings and swine meat on the microbial community of final products, as proposed by Bedia et al. [25].

Although devoid of significative correlations (data not shown), microbial counts of Total Coliforms suggested a relevant role of casing on microbial communities. Indeed, it is worth noting that for all salami, coliforms had initial high values, which decrease significantly (*p* < 0.01) throughout the ripening period. This result could be explained because of the susceptibility of coliforms to the acidification effect of LAB [26,27]. Such finding is in agreement with the Pearson’s correlation coefficients calculated (*R^2^*: −0.446; *p* < 0.01).

Regarding *Pseudomonas* spp., microbiological counts significantly differed among salami and sampling time. Nevertheless, these bacteria followed pH variations showing a significant decrease (*p* < 0.01) at 30 days of maturations, when matrices acidification were highest.

Significant differences among salami were detected for yeasts and molds, which were unusually found in casings. At the beginning of the process, these populations appeared higher in S75 than the others (*p* < 0.01). On the contrary, during the ripening period values came closer. Yeasts and molds had similar growth trends with characteristic bell-shaped. Indeed, since the sixteenth day, their number decreased and approached the initial values. As reported by several authors [27,28], the presence of yeasts is desirable because they contribute to providing salami sausages with their peculiar flavors and surface appearance, because of their esterification and proteolytic activities [29].

To monitor the changes in microbial communities after ripening, it was interesting to highlight the behavior of the investigated population. In particular, the ripening process and manufactories adopted might be considered able to control the hygienic state of products, influencing the survival of microorganisms such as Coliforms.

### 3.2. pH and a_w_ Measurements

The average weight loss of all salami was 40% compared to the initial weight. No significant differences were found among the types of salami. During ripening, pH showed a similar trend among the different salami with a strong decrease until the first month and a slight increase from T1 to T3, as observed in other studies [22,30]. Nevertheless, all products showed pH values higher than 5.2 which was considered the stability point of meat products by Ambrosiadis et al. [31]. Variation in the bacterial population during salami fermentation appeared to influence the meat sourness, as demonstrated by several authors (i.e., [32]). At the beginning of the study (day 1), the pH was 5.89 ± 0.12 in all samples, reaching values of 5.13 ± 0.04 due to acidification activity of Lactic Acid Bacteria. Instead, a negative and significant correlation was found between LAB concentration and pH value, along whole storage time and in each sample (*R^2^*: −0.510; *p* < 0.01). The progressive increase in pH was probably due to proteolytic activities induced by typical meat bacteria with the release of the amino groups [33,34], responsible for pH neutralization [34]. Moreover, Mendonça et al. [35] hypothesized that it could be influenced by the degradation of lactic acid by molds and internal yeasts.

Significant differences (*p* < 0.01) were observed between RS and S75 along the whole storage time (Table 1), with the lowest values in S75 samples. In spite of the content of Apulo-Calabrese meat, the pH values of S50 salami did not appear influenced by indigenous Italian pig breed and almost overlapped to RS. Aboagye et al. [36] compared meat quality traits of Apulo-Calabrese with respect to crossbreeds [Duroc × (Landrace × Large White)] and observed that pH values were constantly higher in autochthonous breed meat, albeit slightly.

Water activity measured at the start of the drying process (1 day) was approximately 0.977 ± 0.001 in all samples and decreased during ripening. Throughout the first three months, each sample showed similar a_w_ levels, underlining the same downward trend. Slight differences have been detected at 120 days: white pork salami (RS) reached a value of 0.794 ± 0.008, lower than the others. This may be related to the possible differences in salt concentration among the three formulations.

### 3.3. Fatty Acids Analysis

Fatty acid composition results measured for each FAME sample in three formulations (SR, S50 and S75) during ripening are shown in Table 2.

Gas chromatography (GC) analysis showed that in all formulations the dominant fatty acids found were palmitic acid (C16:0), stearic acid (C18:0), oleic acid (C18:1n-9), and linoleic acid (C18:2n-6). As a consequence of the different content of black pig meat, significant differences among the three types of salami were highlighted. The average values of total saturated FA throughout the ripening time were 42.27% in SR, 39.02% in S50, and 36.56% in S75. For monounsaturated short- and medium-chain fatty acids, oleic acid was the most abundant (46.62% in SR, 49.52% in S50, and 51.66% in S75, expressed as average values of all sampling times). A higher amount of SFAs were detected in SR samples (*p* < 0.01) than S75 ones, due to greater content in C16:0 and C18:0. According to several authors [2,3,4], Italian native pigs such as *Cinta Senese*, *Casertana*, and *Nero Siciliano* showed a high predisposition to MUFAs depot, mainly oleic, detected in final products as fresh subcutaneous fat and salami products. The high content of C18:1n-9 in salami with Apulo-Calabrese meat (S50 and S75) could be due to the rearing system of the local breeds (almost always outdoors) and the typical dietary regimen of these pigs, which are fed on acorns and chestnuts. Although comparisons with similar studies are difficult due to differences in rearing and management conditions (factors affecting the fatty acids composition of tissues). The high content of oleic acid in meat products from pigs fed on acorns has been reported both by Pugliese et al. [37] in *Cinta Senese* and by Pérez-Palacios et al. [38] in Iberian pigs. Local pig breeds generally exhibit a higher percentage of MUFAs than crossbreeds [2,3,4,5], which are characterized by a greater amount of SFAs was found [2]. This implication is very important from a nutritional viewpoint because stearic and palmitic acids are the predominant SFAs in animal fats whose consumption in the human diet is one of the factors causing cardiovascular disease [39].

The high PUFAs content may have detrimental effects on the sensory and technological quality [40] and acceptability of meat products [41]. The results showed that the lowest PUFAs content was found in SR as the linoleic acid content was significantly lower (*p* < 0.01) than in S50 and S75 from the first to the 60th day of ripening. Yu et al. [42] described similar differences among different genotypes in *longissimus dorsi* muscle, recording significantly more PUFAs in Lantang (IMF 2.46%) than in Landrase (IMF 1.43%) breeds. Nevrkla et al. [5] found the same difference in backfat samples of Prestice Black-Pied breed (IMF 2.89%) than in pigs of hybrid combination [Large White × Landrace sows × Duroc × Pietrain] (IMF 1.99%). In contrast, Aboagye et al. [17] comparing the fatty acid profile of Apulo-Calabrese and crossbreed pigs reared indoors and fed the same commercial diet, observed significantly lower PUFAs content in the Italian local breed. However, it is difficult to compare studies because the difference in PUFAs content may be due to the different growth performance and adipogenic potential that characterize the different genetic types considered.

In long-aged salami, the PUFAs level should not exceed 12% of the total fatty acids [43]. At the start of ripening, the percentage of PUFAs was approximately 11–12 in all samples. During ripening, the PUFAs content is more susceptible to oxidation reactions than saturated and monounsaturated fatty acids [44]. PUFAs decreased in all samples except in SR. SFAs content gradually decreased during the first 60 days and then increased at the end of ripening. In contrast, MUFAs percentage increased during the first 60 days and then suffered a fall at the end of ripening. Regarding P/S, a value greater than 0.4 is recommended for healthy foods and diets [45,46] in order to prevent both an excess of SFAs, having a negative effect on the LDL cholesterol plasmatic level, and of PUFAs, some of which are precursors of powerful clotting agents and are involved in the etiology of some cancers [47].

The PUFA/SFA ratio (P/S), the MUFA/SFA ratio (M/S), the hypocholesterolemic/Hypercholesterolemic fatty acids ratio (h/H), Atherogenic index (AI), and Thrombogenic index (TI) were determined (Table 3).

The P/S ratio was below the recommended value and ranged on average from 0.26 in SR to 0.31 in S75. The high percentages of MUFAs and C18:1n-9 in S75 samples indicate their suitability for healthier diets, as diets rich in MUFAs (and PUFAs) reduce blood cholesterol levels and are related to a low incidence of cardiovascular diseases [11]. The M/S ratio was the highest in S75 on the 60th day due to the highest content of oleic acid (47.8%). On the contrary, SR showed the lowest ratio M/S at the end of ripening caused by the lowest percentage of MUFAs, characterized by 41.7% oleic acid.

Among SFAs, C18:0 has poor atherogenic characteristics because it is rapidly desaturated in oleic acid, unlike myristic acid (C14:0) which is considered the main atherogenic fatty acid as it has a hypercholesterolemic power four times higher than C16:0 [48]. Ulbricht and Southgate [18] proposed equations for atherogenic and thrombogenic indices. Therefore, the atherogenic index, as well as the thrombogenic index, were calculated in order to evaluate the risk of atherosclerosis, and the potential aggregation of blood platelets, respectively. TI and AI ranged from 1.38 to 1.08 and from 0.55 to 0.44 (mean values) in SR and S75, respectively. The average values for S75 were consistent with the results by Franci et al. [3] for Cinta Senese. AI and TI values for SR were similar to the values found by Del Nobile et al. [49] for commercial salami. Our results allow us to consider salami made with black pigs healthier than the salami SR.

These observations agree with the values of the h/H ratio, which is also used to evaluate the nutritive quality of the fat as it is related to cholesterol metabolism [19]. From a nutritional standpoint, higher HH values are considered more beneficial for human health. The percentage of fatty acids considered as hypocholesterolemic was significantly higher in the S75 (>63%) than in the SR (<58%), while the amount of Hypercholesterolemic fatty acids showed an opposite behavior (<24% in the S75 vs. >26% in the SR). As a result, the mean value of the h/H ratio of the S75 was significantly favorable (>2.6) in particular on the 60th day of ripening (>2.7).

### 3.4. Rheological Analysis

Significant differences were observed as a function of the meat batch used for salami production and the ripening time.

#### 3.4.1. Texture Profile Analysis

The texture profile analysis (TPA) results are reported in Table 4.

Hardness and gumminess gradually increased in different salami formulations. The highest (*p* < 0.01) absolute variations occurred during the longest interval (60–120 days). This increase could be due to the coagulation of protein at low pH and the decrease in the moisture content as reported by Bozkurt et al. [50]. S75 showed the lowest hardness in all sampling times compared to the other two formulations (SR and S50). According to Wood et al. [46], the lower hardness could be attributable not only to the higher fat content but, in particular, to fatty acid composition. The effect of fatty acids on hardness is due to their different melting points [46] that are influenced by the degree of unsaturation. Maw et al. [51] showed that the increase in softness was associated with an increase in linoleic acid and a decrease in stearic and palmitic acid content. The Pearson’s correlation test (Table 5) indicated that there is a positive significant relationship (*p* < 0.05) between hardness and palmitic acid and a negative significant relationship (*p* < 0.05) between hardness and linoleic acid. Furthermore, variations in the molecule structure are important: *cis* fatty acids have lower melting points than *trans* isomers [46]. In the present study, the percentage of *cis* fatty acid, such as oleic acid, showed a negative significant correlation (*p* < 0.05) with hardness. This result confirms the lowest hardness value of S75: in this formulation, hardness was influenced by the lowest palmitic acid percentage and by the highest oleic acid percentage compared to SR and S50. As reported by Bañón et al. [26], an increase in hardness corresponds to a decrease in springiness and cohesiveness. These values decreased during the ripening period, but the highest (*p* < 0.01) variations occurred during the longest interval (60–120 days). Resilience values did not change (*p* > 0.05) from the 30th to the 120th day. Adhesiveness values decreased significantly (*p* < 0.01) during ripening, in particular S50 seems to have become easily sliceable as it showed the highest value of adhesiveness at the end of ripening.

#### 3.4.2. Color

The results of color measurements (L*, a*, b*, Chroma, and Hue angle value) are reported in Table 6.

SR had lower L* values (*p* < 0.01) than S75 on the 30th and the 120th day of ripening. A lower SR lipid content could explain the difference in L* value because fat grains contribute to the light reflection of slices and the lean meat can make color determination more difficult due to nitrous myoglobin formation. Furthermore, brightness seems to be related to stearic and oleic acids as reported by several authors [52,53]. Ventanas et al. [52] in a study to evaluate the effects of a specific diet on characteristics of a cured ham, identified a relationship between chemical and sensory parameters and showed the opposite role that two fatty acids have on the L* parameter. In accord with our results, although stearic acid showed no significant correlation (*p* > 0.05) with brightness, oleic and palmitic acids played a principal role in some appearance traits. Oleic acid showed a positive significant correlation (*p* < 0.01) with brightness while palmitic acid was negatively correlated (*p* < 0.01) to brightness (Table 6).

These results seem to confirm that palmitic and oleic acids negatively affected the brightness in the S50; on the contrary, their content positively influenced it in the S75. In all formulations the L* value remained stable with a decrease at the last sample interval (from the 60th to the 120th day). This decrease could be caused by the dark coloration due to the browning reaction [54]. Similarly, Bañón et al. [26] observed that the L* value of salami decreased during the 120 days of ripening. On the 30th day, the b* value was lower (*p* < 0.05) in S75 than in SR and S50. The observed color changes were mainly attributable to a transformation of the muscle pigment linked to the nitrite ion. The a* value was lower (*p* < 0.01) in SR than in S50 and S75 since the 60th day: this can be explained by the total or partial denaturation of nitrous myoglobin as a result of lactic acid production [54]. However, even if a* and b* values reflected the same variations of L* value, a definite trend was not evaluated. The S75 was brighter, and its color spectrum tended more towards red than the other two salami formulations (SR and S50) and also showed the highest *Chroma* value on the 60th day of ripening. At the same time, since the *Hue angle* is considered a measure of color change appreciable by the human eye, S75 was characterized by a less stable color. However, as discussed above, this instability is due to an increase in a* values and, therefore, the small hue angle values collected in S75 up to the 60th day of ripening indicate the development of the color towards red space during storage. These differences in color could be mainly due to differences in the meat pigmentation of the raw mixture. In agreement with Aboagye et al. [36], Apulo-Calabrese meats have a deeper red color than other local European pig breeds [3]. Furthermore, Bedia et al. [55] showed that additive-free salami produced from rustic pigs were redder than salami from commercial crossbreed.

### 3.5. Thiobarbituric Acid-Reactive Substances (TBARS)

Lipid oxidation is one of the main factors affecting the features of meat products during the storage period. The development of oxidation products in salami is displayed in Table 7 as TBARS values.

Oxidation significantly increased along the ripening process for all samples (*p* < 0.01), except for S75 in the last sampling time. It is worth noting that since the day after manufactories salami SR showed the lowest concentration of malondialdehyde (MDA—0.042 mg/Kg), almost half of the other two formulations (*p* < 0.01). Therefore, the presence of Apulo-Calabrese meat seems to play a role in the oxidation process, especially considering MDA differences among S50 and S75 which were clearly influenced by the percentage of indigenous meat present. Other authors demonstrated that lipid oxidation depends on polyunsaturated fatty acids contents, probably because of the lower energy necessary to remove hydrogen from a double carbon bond than methyl carbon, especially when the carbon is between two double bonds [44,56]. Nonetheless, PUFAs contents were higher in S75 than others, no significant correlations (data not shown) were found between PUFAs contents and TBARS values. Differences among products remained almost similar until the 16th day of the ripening process, while at the fourth-month disparities disappeared and a significant increase in the MDA values was observed in SR and S50. In particular, the lowest TBARS value was measured in S75 (0.264 mg/kg), probably due to the transformation of oxidation indicators into other decomposition products, afterword achieving its peak [57]. Furthermore, Pavlović et al. [58] stated that lipid oxidation slowdown is due to interactions between MDA and additives and organics molecules, as carbohydrates and amino acids.

Table 5 shows the Pearson’s correlation coefficients between TBARS vs. sensory features. Since lipid oxidation was liked to decrease in redness, due to its role as a promoter of myoglobin oxidation, no significant correlation was found. Surprisingly, the value of brightness was negatively correlated (*p* < 0.01) with the oxidation lipid index. Though positive correlation was found among TBARS level and hardness (*p* < 0.01), because of the common link to high fat content.

## 4. Conclusions

The reassessment of the link between swine breeds and typical foods might be an important tool to increase the farming value and avoid its decline. Innovative health-focused products are very attractive for consumers who want to savor new flavors without creating imbalances in their diet and causing damage to their health. In this regard, some authors have already suggested that the partial inclusion of autochthonous breed meat allows to improve the quality of different products, and our results uphold them.

Overall, our study aimed to combine manufactories and ripening phase to evaluate the whole process as a possible value added to local production. As known, ripening strongly influences the characteristics of final dry fermented pork products, therefore, it might be considered as a critical phase. Results pointed out that the partial inclusion of Apulo-Calabrese meat in meat products influenced fatty acid profile, lipid oxidation, and rheological parameters of salami. The binding agent could be the higher level of fat in salami produced with Apulo-Calabrese meat with respect to commercial ones. Overall, data showed clearly that the best combination among ripening time and chemical compositions has been reached in S75 samples on the 60th day. Differences in swine meat and fat content did not condition microbiological analyses, instead, we believe it is appropriate to underline the negative burden of natural casings on microbiological communities of products. However, the ripening system was capable of tearing down coliforms which were replaced by lactic acid bacteria.

This study points out the potential role of autochthonous animals in the enhancement of local productions that could be a source of products capable of satisfying a very broad target of consumers if only data relating to atherogenic and thrombogenic indices are considered. However, it is worth noting that the obvious quality of native breed products can be lost if comparable attention to the process is not applied. The choice of suitable aging processes and specific production techniques seems to occupy a fundamental rule because it is able to maintain the productivity of the salami all year round.

The cost related to local breed farming and the lower slaughter weight compared to hybrid breeds must be considered in order to find the right balance between costs and benefits. The S50 formulation appeared a suitable choice that might satisfy consumers for nutritional expectations and sensory profiles, and producers for its lower expensiveness.

## Figures and Tables

**Table 1:** Microbiological and physicochemical results.

	Day	1	30	60	120
		M ± sE	M ± sE	M ± sE	M ± sE
TAB 30 °C	BD	5.62 ± 0.18 ^X^			
	SR	4.96 ± 0.17 ^Y,A^	6.51 ± 0.22 ^B^	8.12 ± 0.17 ^X,C^	7.86 ± 0.17 ^xX,D^
	S50	5.2 ± 0.17 ^Z,A^	6.30 ± 0.16 ^B^	8.39 ± 0.17 ^Y,C^	8.24 ± 0.24 ^yX,C^
	S75	5.78 ± 0.16 ^X,A^	6.22 ± 0.39 ^aB^	7.18 ± 0.19 ^Z,bB^	9.22 ± 0.19 ^Y,C^
Total coliforms	BD	5.26 ± 0.12 ^X^			
	SR	4.36 ± 0.14 ^Y,A^	3.37 ± 0.12 ^X,B^	2.97 ± 0.12 ^X,C^	1.39 ± 0.12 ^X,D^
	S50	4.19 ± 0.11 ^Y,A^	3.74 ± 0.13 ^Y,B^	3.33 ± 0.12 ^Y,C^	1.95 ± 0.11 ^Y,D^
	S75	4.15 ± 0.10 ^Y,A^	3.43 ± 0.20 ^B^	4.44 ± 0.12 ^Z,C^	1.40 ± 0.14 ^X,D^
Lactic acid bacilli	BD	4.19 ± 0.13 ^xX^			
	SR	3.60 ± 0.16 ^Y,A^	5.94 ± 0.13 ^X,B^	7.49 ± 0.13 ^X,C^	7.76 ± 0.19 ^X,C^
	S50	4.01 ± 0.13 ^yX,A^	4.61 ± 0.15 ^Y,B^	8.42 ± 0.19 ^Y,C^	7.52 ± 0.18 ^X,D^
	S75	4.56 ± 0.17 ^Z,A^	4.66 ± 0.14 ^Y,A^	8.82 ± 0.14 ^Y,B^	6.62 ± 0.17 ^Y,C^
*Pseudomonas* spp.	BD	5.25 ± 0.10 ^X^			
	SR	3.18 ± 0.11 ^Y,A^	1.34 ± 0.11 ^X,B^	3.57 ± 0.12 ^X,C^	4.15 ± 0.11 ^X,D^
	S50	5.20 ± 0.13 ^X,A^	2.83 ± 0.13 ^Y,B^	3.62 ± 0.16 ^X,C^	5.15 ± 0.11 ^Y,A^
	S75	5.70 ± 0.13 ^Z,A^	2.95 ± 0.10 ^Y,B^	2.91 ± 0.10 ^Y,B^	3.48 ± 0.16 ^Z,C^
Yeasts	BD	2.84 ± 0.13 ^X^			
	SR	3.17 ± 0.12 ^xY,A^	4.83 ± 0.15 ^x,B^	5.63 ± 0.15 ^X,C^	3.93 ± 0.15 ^D^
	S50	3.40 ± 0.14 ^yY,A^	4.74 ± 0.17 ^B^	4.84 ± 0.14 ^Y,B^	4.14 ± 0.17 ^C^
	S75	3.85 ± 0.13 ^Z,A^	4.43 ± 0.17 ^y,B^	4.90 ± 0.14 ^Y,C^	3.88 ± 0.13 ^A^
Molds	BD	2.56 ± 0.10 ^xX^			
	SR	2.78 ± 0.12 ^yX,A^	4.84 ± 0.16 ^X,B^	4.82 ± 0.10 ^B^	3.69 ± 0.10 ^xX,C^
	S50	3.21 ± 0.09 ^Y,A^	4.87 ± 0.11 ^X,B^	4.78 ± 0.16 ^B^	3.85 ± 0.11 ^yX,C^
	S75	3.88 ± 0.10 ^Z,A^	5.43 ± 0.10 ^Y,B^	4.79 ± 0.16 ^C^	4.39 ± 0.10 ^Y,D^
pH	SR	5.97 ± 0.01 ^X,A^	5.17 ± 0.01 ^X,B^	5.29 ± 0.03 ^X,C^	5.54 ± 0.00 ^X,D^
	S50	5.96 ± 0.02 ^X,A^	5.08 ± 0.01 ^Y,B^	5.18 ± 0.02 ^xY,C^	5.51 ± 0.01 ^Y,D^
	S75	5.75 ± 0.03 ^Y,A^	5.13 ± 0.00 ^Z,B^	5.11 ± 0.03 ^yY,B^	5.29 ± 0.01 ^Z,C^
a_w_	SR	0.978 ± 0.002 ^A^	0.837 ± 0.013 ^B^	0.848 ± 0.009 ^B^	0.794 ± 0.008 ^X,C^
	S50	0.976 ± 0.001 ^x,A^	0.863 ± 0.004 ^X,B^	0.854 ± 0.005 ^B^	0.835 ± 0.004 ^Y,C^
	S75	0.978 ± 0.000 ^y,A^	0.825 ± 0.003 ^Y,aB^	0.849 ± 0.006 ^C^	0.837 ± 0.005 ^Y,bC^

In each sampling day, three samples by type of salami were analyzed. Statistical analysis was performed comparing experimental groups at each sampling time and within each experimental group along the ripening period. All data were presented as the least square mean (M) ± standard error (sE). Different superscript uppercase letters indicate a significant difference at *p* < 0.01. Different superscript lowercase letters indicate a significant difference at *p* < 0.05. ^a–d^ Mean values in the same row (same batch in different weeks) with different letters presented significant differences. ^x–z^ Mean values in the same column (different samples on the same ripening time) with different letters presented significant differences.

**Table 2:** Fatty acid composition (%) of three salami (SR, S50, and S75) during ripening.

	Day	1	30	60	120
		M ± sE	M ± sE	M ± sE	M ± sE
C14:0	SR	1.76 ± 0.02 ^X,a,A^	1.68 ± 0.03 ^X,b^	1.51 ± 0.06 ^X,a,B^	1.81 ± 0.04 ^X,a,A^
	S50	1.69 ± 0.01 ^Y,A^	1.52 ± 0.02 ^Y,B^	1.44 ± 0.04 ^X,a,B^	1.56 ± 0.01 ^Y,b,B^
	S75	1.44 ± 0.04 ^Z,A^	1.40 ± 0.02 ^Z,A^	1.26 ± 0.04 ^Y,B^	1.33 ± 0.01 ^Z,B^
C16:0	SR	24.26 ± 0.04 ^X,A^	25.44 ± 0.05 ^X,B^	24.24 ± 0.14 ^X,A^	26.04 ± 0.04 ^X,B^
	S50	23.38 ± 0.04 ^Y,A^	24.16 ± 0.02 ^Y,a,B^	23.13 ± 0.04 ^Y,C^	24.06 ± 0.04 ^Y,b,B^
	S75	22.00 ± 0.04 ^Z,A^	22.57 ± 0.04 ^Z,B^	21.71 ± 0.06 ^Z,C^	22.33 ± 0.03 ^Z,D^
C18:0	SR	15.58 ± 0.02 ^X,A^	14.48 ± 0.16 ^X,B^	15.10 ± 0.09 ^X,C^	15.10 ± 0.09 ^X,D^
	S50	13.27 ± 0.20 ^Y,a^	12.80 ± 0.07 ^Y,b^	12.79 ± 0.03 ^Y,b^	12.84 ± 0.04 ^Y,b^
	S75	12.61 ± 0.03 ^Z,a,A^	11.76 ± 0.01 ^Z,a,B^	12.15 ± 0.15 ^Z,b,C^	12.31 ± 0.12 ^Z,b,C^
C16:1	SR	2.24 ± 0.03 ^x,X,a^	2.16 ± 0.01 ^X,b^	2.25 ± 0.06	2.28 ± 0.09
	S50	2.55 ± 0.07 ^Y,A^	2.26 ± 0.02 ^Y,a,B^	2.17 ± 0.02 ^A,B^	2.46 ± 0.09 ^b,A^
	S75	2.33 ± 0.03 ^y,X,A^	2.27 ± 0.01 ^Y,a,A^	2.11 ± 0.05 ^B^	2.42 ± 0.07 ^b,A^
C18:1n9c	SR	41.97 ± 0.13 ^X,A^	42.18 ± 0.10 ^X,A^	43.22 ± 0.17 ^X,a,B^	41.70 ± 0.61 ^X,b^
	S50	44.36 ± 0.11 ^Y,A^	45.46 ± 0.01 ^Y,a,B^	45.40 ± 0.03 ^Y,b,B^	45.72 ± 0.02 ^Y,C^
	S75	46.72 ± 0.04 ^Z,A^	47.65 ± 0.10 ^Z,B^	47.83 ± 0.08 ^Z,B^	46.54 ± 0.09 ^Z,A^
C18:1n9t	SR	1.05 ± 0.03 ^x,A^	1.37 ± 0.01 ^X,a,B^	1.23 ± 0.04 ^X,C^	1.12 ± 0.10 ^x,X,b^
	S50	0.94 ± 0.04 ^y,A^	0.89 ± 0.02 ^Y,A^	0.93 ± 0.00 ^Y,A^	0.63 ± 0.03 ^Y,B^
	S75	0.96 ± 0.03 ^y,a,A^	0.83 ± 0.06 ^Y,b,A^	0.79 ± 0.02 ^Z,B^	1.48 ± 0.09 ^y,X,C^
C20:1n9	SR	0.45 ± 0.01 ^A^	0.50 ± 0.01 ^x,B^	1.01 ± 0.02 ^X,C^	0.76 ± 0.16
	S50	0.47 ± 0.02 ^A^	0.52 ± 0.02 ^B^	1.64 ± 0.05 ^Y,A^	0.49 ± 0.02 ^X,B^
	S75	0.48 ± 0.02 ^A^	0.55 ± 0.02 ^y,B^	1.38 ± 0.04 ^Z,C^	1.04 ± 0.01 ^Y,D^
C18:2n6c	SR	10.53 ± 0.18 ^X,A^	10.23 ± 0.00 ^X,A^	9.41 ± 0.03 ^X,B^	10.14 ± 0.48
	S50	11.33 ± 0.11 ^Y,A^	10.30 ± 0.04 ^X,B^	10.25 ± 0.09 ^Y,B^	9.63 ± 0.12 ^C^
	S75	11.43 ± 0.06 ^Y,A^	10.77 ± 0.05 ^Y,B^	10.45 ± 0.26 ^Y,a,B^	9.87 ± 0.03 ^b,C^
C18:3n3	SR	0.31 ± 0.03 ^a,A^	0.32 ± 0.02 ^x,A^	0.41 ± 0.01 ^X,B^	0.47 ± 0.05 ^x,b,B^
	S50	0.31 ± 0.03 ^a,A^	0.38 ± 0.00 ^y,b,A^	0.49 ± 0.03 ^Y,B^	0.60 ± 0.01 ^y,X,C^
	S75	0.32 ± 0.02 ^A^	0.36 ± 0.07 ^a^	0.46 ± 0.01 ^Y,B^	0.53 ± 0.02 ^y,Y,b,C^
C20:4n6	SR	0.53 ± 0.01 ^x^	0.54 ± 0.01 ^x,a^	0.54 ± 0.01 ^X,a^	0.47 ± 0.03 ^x,b^
	S50	0.51 ± 0.01 ^y,A^	0.56 ± 0.02 ^X,a^	0.58 ± 0.01 ^Y,B^	0.49 ± 0.03 ^x,b,A^
	S75	0.52 ± 0.01 ^A^	0.64 ± 0.02 ^y,Y,a,B^	0.65 ± 0.02 ^Z,a,B^	0.57 ± 0.03 ^y,b^
ΣSFA	SR	42.44 ± 0.10 ^X,A^	42.39 ± 0.09 ^X,a,A^	41.61 ± 0.18 ^X.B^	42.66 ± 0.09 ^X,b,A^
	S50	39.22 ± 0.17 ^Y,A^	39.30 ± 0.07 ^Y,A^	38.16 ± 0.09 ^Y,B^	39.43 ± 0.05 ^Y,A^
	S75	36.93 ± 0.02 ^Z,A^	36.53 ± 0.05 ^Z,a,B^	35.93 ± 0.21 ^Z,b,B^	36.88 ± 0.11 ^Z,A^
ΣMUFA	SR	45.95 ± 0.08 ^X,A^	46.47 ± 0.12 ^X,B^	47.96 ± 0.17 ^X,a,C^	46.13 ± 0.80 ^X,b^
	S50	48.55 ± 0.23 ^Y,A^	49.40 ± 0.04 ^Y,B^	50.42 ± 0.05 ^Y,C^	49.72 ± 0.07 ^Y,D^
	S75	50.72 ± 0.05 ^Z,A^	51.62 ± 0.03 ^Z,a,B^	52.43 ± 0.03 ^Z,C^	51.90 ± 0.11 ^Z,b,B^
ΣPUFA	SR	11.45 ± 0.14 ^X,a,A^	11.15 ± 0.01 ^X,b,A^	10.43 ± 0.06 ^X,B^	11.29 ± 0.59
	S50	12.23 ± 0.07 ^Y,A^	11.30 ± 0.01 ^Y,B^	11.42 ± 0.03 ^Y,C^	10.86 ± 0.10 ^X,D^
	S75	12.35 ± 0.08 ^Y,a,A^	11.85 ± 0.08 ^Z,B^	11.64 ± 0.23 ^Y,b^	11.19 ± 0.02 ^Y,C^
n-3	SR	0.31 ± 0.03 ^a,A^	0.32 ± 0.02 ^x,A^	0.41 ± 0.01 ^X,B^	0.47 ± 0.05 ^x,b,B^
	S50	0.31 ± 0.03 ^a,A^	0.38 ± 0.00 ^y,b,A^	0.49 ± 0.03 ^Y,B^	0.60 ± 0.01 ^y,X,C^
	S75	0.32 ± 0.02 ^A^	0.36 ± 0.07 ^a^	0.46 ± 0.01 ^Y,B^	0.53 ± 0.02 ^Y,b,C^
n-6	SR	11.13 ± 0.17 ^X,A^	10.83 ± 0.01 ^X,A^	10.02 ± 0.05 ^X,B^	10.82 ± 0.54
	S50	11.92 ± 0.10 ^Y,A^	10.92 ± 0.01 ^Y,B^	10.93 ± 0.06 ^Y,B^	10.26 ± 0.09 ^X,C^
	S75	12.03 ± 0.06 ^Y,A^	11.49 ± 0.02 ^Z,B^	11.18 ± 0.22^Y,a,B^	10.66 ± 0.04 ^Y,b,C^

In each sampling day, three samples by type of salami were analyzed. Statistical analysis was performed comparing experimental groups at each sampling time and within each experimental group along the ripening period. All data were presented as the least square mean (M) ± standard error (sE). Different superscript uppercase letters indicate a significant difference at *p* < 0.01. Different superscript lowercase letters indicate a significant difference at *p* < 0.05. ^a–d^ Mean values in the same row (same batch in different weeks) with different letters presented significant differences. ^x–z^ Mean values in the same column (different samples on the same ripening time) with different letters presented significant differences.

**Table 3:** Nutritional indices and the fatty acid ratio of three salami (SR, S50, and S75) during ripening.

	Day	1	30	60	120
		M ± sE	M ± sE	M ± sE	M ± sE
M/S	SR	1.08 ± 0.00 ^X,a,B^	1.10 ± 0.01 ^X,b,A^	1.15 ± 0.01 ^X,B^	1.08 ± 0.02 ^X,A^
	S50	1.24 ± 0.01 ^Y,A^	1.26 ± 0.00 ^Y,A^	1.32 ± 0.01 ^Y,B^	1.26 ± 0.00 ^Y,A^
	S75	1.14 ± 0.23	1.41 ± 0.00 ^Z,A^	1.46 ± 0.01 ^Z,B^	1.41 ± 0.01 ^Z,A^
P/S	SR	0.27 ± 0.00 ^X,A^	0.26 ± 0.00 ^X,A^	0.25 ± 0.00 ^X,B^	0.27 ± 0.01 ^X^
	S50	0.31 ± 0.00 ^Y,A^	0.29 ± 0.00 ^Y,B^	0.30 ± 0.00 ^Y,C^	0.28 ± 0.00 ^X,D^
	S75	0.28 ± 0.05	0.32 ± 0.00 ^Z,A^	0.32 ± 0.01 ^Z,a^	0.30 ± 0.00 ^Y,b,B^
Ai	SR	0.55 ± 0.00 ^X,A^	0.56 ± 0.00 ^X,B^	0.52 ± 0.00 ^X,C^	0.58 ± 0.01 ^X,D^
	S50	0.50 ± 0.00 ^Y,A^	0.50 ± 0.00 ^Y,A^	0.47 ± 0.00 ^Y,B^	0.50 ± 0.00 ^Y,A^
	S75	0.44 ± 0.00 ^Z,A^	0.45 ± 0.00 ^Z,a,A^	0.42 ± 0.00 ^Z,B^	0.44 ± 0.00 ^Z,b,A^
Ti	SR	1.41 ± 0.00 ^X,A^	1.40 ± 0.00 ^X,a,A^	1.35 ± 0.01 ^X,B^	1.39 ± 0.00 ^X,b,C^
	S50	1.23 ± 0.01 ^Y,A^	1.23 ± 0.00 ^Y,a,A^	1.16 ± 0.01 ^Y,B^	1.21 ± 0.01 ^Yb,A^
	S75	1.11 ± 0.01 ^Z,a,A^	1.09 ± 0.01 ^Z,b,A^	1.06 ± 0.01 ^Z,B^	1.09 ± 0.01 ^Z,b,A^
h/H	SR	2.21 ± 0.01 ^X,A^	2.13 ± 0.00 ^X,B^	2.27 ± 0.01 ^X,C^	2.06 ± 0.01 ^X,D^
	S50	2.42 ± 0.01 ^Y,A^	2.36 ± 0.00 ^Y,B^	2.52 ± 0.01 ^Y,C^	2.36 ± 0.01 ^Y,B^
	S75	2.69 ± 0.02 ^Z,a,A^	2.65 ± 0.00 ^Z,b,A^	2.79 ± 0.01 ^Z,B^	2.67 ± 0.01 ^Z,A^

In each sampling day, three samples by type of salami were analyzed. Statistical analysis was performed comparing experimental groups at each sampling time and within each experimental group along the ripening period. All data were presented as the least square mean (M) ± standard error (sE). Different superscript uppercase letters indicate a significant difference at *p* < 0.01. Different superscript lowercase letters indicate a significant difference at *p* < 0.05. ^a–d^ Mean values in the same row (same batch in different weeks) with different letters presented significant differences. ^x–z^ Mean values in the same column (different samples on the same ripening time) with different letters presented significant differences.

**Table 4:** Texture profile analysis (TPA) of three salami (SR, S50, and S75) during ripening.

	Day	30	60	120
		M ± sE	M ± sE	M ± sE
Hardness	SR	31.13 ± 1.50 ^X,A^	35.07 ± 2.76 ^x,A^	70.05 ± 3.30 ^X,B^
	S50	30.30 ± 1.24 ^X,A^	32.16 ± 2.09 ^A^	62.43 ± 2.62 ^X,B^
	S75	21.13 ± 1.05 ^Y,A^	29.29 ± 0.90 ^y,B^	44.31 ± 2.62 ^Y,C^
Cohesiveness	SR	0.46 ± 0.03	0.45 ± 0.02 ^X^	0.45 ± 0.02 ^x^
	S50	0.47 ± 0.02	0.50 ± 0.02	0.47 ± 0.01
	S75	0.54 ± 0.10	0.54 ± 0.01 ^Y,A^	0.43 ± 0.01 ^y,B^
Springiness	SR	0.97 ± 0.04 ^A^	0.90 ± 0.01 ^X,A^	0.86 ± 0.01 ^x,B^
	S50	1.03 ± 0.05 ^A^	0.84 ± 0.02 ^Y,B^	0.93 ± 0.02 ^y,A^
	S75	1.01 ± 0.06 ^a^	0.87 ± 0.02 ^b^	0.90 ± 0.02 ^y^
Gumminess	SR	14.19 ± 0.92 ^A^	16.13 ± 1.76 ^A^	31.49 ± 1.94 ^X,B^
	S50	14.20 ± 1.00 ^A^	15.82 ± 0.95 ^A^	29.14 ± 1.66 ^X,B^
	S75	11.33 ± 2.02 ^a,A^	15.75 ± 0.65 ^b,A^	19.23 ± 1.50 ^Y,B^
Chewiness	SR	13.60 ± 0.73 ^A^	14.45 ± 1.58 ^A^	26.98 ± 1.66 ^X,B^
	S50	14.36 ± 0.86 ^A^	13.22 ± 0.73 ^A^	26.77 ± 1.38 ^X,B^
	S75	11.15 ± 1.80 ^A^	13.77 ± 0.62 ^a^	17.36 ± 1.43 ^Y,b,B^
Adhesiveness	SR	−2.03 ± 0.57 ^X,A^	−3.38 ± 0.72 ^x,A^	−10.18 ± 2.12 ^X,B^
	S50	−6.44 ± 1.26 ^Y,A^	−1.75 ± 0.46 ^B^	−18.01 ± 1.47 ^Y,C^
	S75	−4.35 ± 1.12 ^a,A^	−1.74 ± 0.35 ^y,b,A^	−12.09 ± 2.61 ^B^
Resilience	SR	0.29 ± 0.08	0.23 ± 0.03	0.18 ± 0.05
	S50	0.39 ± 0.11 ^a^	0.17 ± 0.03 ^b^	0.17 ± 0.04
	S75	0.36 ± 0.11	0.17 ± 0.02	0.28 ± 0.10

In each sampling day, three samples by type of salami were analyzed. Statistical analysis was performed comparing experimental groups at each sampling time and within each experimental group along the ripening period. All data were presented as the least square mean (M) ± standard error (sE). Different superscript uppercase letters indicate a significant difference at *p* < 0.01. Different superscript lowercase letters indicate a significant difference at *p* < 0.05. ^a–c^ Mean values in the same row (same batch in different weeks) with different letters presented significant differences. ^x–y^ Mean values in the same column (different samples on the same ripening time) with different letters presented significant differences.

**Table 5:** Pearson’s correlation coefficients (*R*^2^) between chemical parameters/oxidation index and sensory features of salami.

Sensory Features	Chemical Composition							Oxidation Index	
	C16:0		C18:0	C18:1n9c		C18:2n6c		TBARS	
*Appearance*									
Redness	−0.233		−0.319	0.208		0.358		0.155	
Brightness	−0.505	**	−0.220	0.500	**	0.044		−0.577	**
Chroma	−0.282		−0.186	0.155		0.190		0.049	
*Texture*									
Hardness	0.485	*	0.188	−0.406	*	−0.440	*	0.783	**
Cohesiveness	−0.292		−0.334	0.345		0.288		−0.107	

Statistical significance: (*) *p* < 0.05; (**) *p* < 0.01.

**Table 6:** Color measurements (CIE L*a*b*) of three salami (SR, S50, and S75) during ripening.

	Day	30	60	120
		M ± sE	M ± sE	M ± sE
L*	SR	43.97 ± 1.04 ^X,A^	44.59 ± 0.12 ^X,A^	35.81 ± 1.94 ^X,B^
	S50	45.21 ± 1.22 ^A^	43.69 ± 0.10 ^Y,a^	39.44 ± 1.70 ^x,b,B^
	S75	46.98 ± 0.21 ^Y,A^	45.53 ± 1.56	43.24 ± 0.11 ^Y,y,B^
a*	SR	12.07 ± 0.26 ^A^	8.78 ± 0.14 ^X,B^	11.61 ± 0.31 ^A^
	S50	11.60 ± 0.13	11.76 ± 0.16 ^Y^	10.61 ± 0.80
	S75	10.33 ± 0.97 ^A^	13.84 ± 0.30 ^Z,B^	12.57 ± 1.42
b*	SR	5.48 ± 1.04 ^x^	6.91 ± 0.55 ^X^	6.03 ± 0.26 ^x^
	S50	4.82 ± 0.55 ^x,A^	8.77 ± 0.20 ^Y,B^	3.81 ± 0.67 ^y,X,A^
	S75	2.79 ± 0.81 ^y,A^	8.17 ± 0.38 ^B^	8.05 ± 0.87 ^Y,B^
Chroma	SR	13.42 ± 0.26 ^x,A^	11.22 ± 0.27 ^X,B^	13.10 ± 0.30 ^A^
	S50	12.61 ± 0.20 ^y,A^	14.67 ± 0.25 ^Y,B^	11.31 ± 0.96 ^A^
	S75	10.76 ± 1.11 ^y,a,A^	16.09 ± 0.36 ^Z,B^	14.93 ± 1.66 ^b^
Hue angle	SR	24.12 ± 4.38 ^a^	37.95 ± 2.54 ^x,b,A^	27.42 ± 1.20 ^X,B^
	S50	22.43 ± 2.40 ^x,A^	36.71 ± 0.30 ^X,B^	19.10 ± 2.23 ^Y,A^
	S75	13.68 ± 3.50 ^y,A^	30.50 ± 1.16 ^y,Y,B^	32.66 ± 0.22 ^Z,B^

In each sampling day, three samples by type of salami were analyzed. Statistical analysis was performed comparing experimental groups at each sampling time and within each experimental group along the ripening period. All data were presented as the least square mean (M) ± standard error (sE). Different superscript uppercase letters indicate a significant difference at *p* < 0.01. Different superscript lowercase letters indicate a significant difference at *p* < 0.05. ^a–b^ Mean values in the same row (same batch in different weeks) with different letters presented significant differences. ^x–z^ Mean values in the same column (different samples on the same ripening time) with different letters presented significant differences.

**Table 7:** Effects of meat species and ripening time on oxidation status of salami.

	Day	1	30	60	120
		M ± sE	M ± sE	M ± sE	M ± sE
TBARS *	SR	0.042 ± 0.001 ^X,A^	0.037 ± 0.004 ^X,A,B^	0.034 ± 0 ^X,B^	0.300 ± 0 ^X,x,C^
	S50	0.070 ± 0.002 ^Y,A^	0.077 ± 0 ^Y,B^	0.085 ± 0.002 ^Y,C^	0.303 ± 0.001 ^X,y,D^
	S75	0.102 ± 0.001 ^Z,A^	0.140 ± 0 ^Z,B^	0.160 ± 0 ^Z,C^	0.264 ± 0 ^Y,D^

* mg MDA/kg muscle. In each sampling day, three samples by type of salami were analyzed. Statistical analysis was performed comparing experimental groups at each sampling time and within each experimental group along the ripening period. All data were presented as the least square mean (M) ± standard error (sE). Different superscript uppercase letters indicate a significant difference at *p* < 0.01. Different superscript lowercase letters indicate a significant difference at *p* < 0.05. ^a–c^ Mean values in the same row (same batch in different weeks) with different letters presented significant differences. ^x–z^ Mean values in the same column (different samples on the same ripening time) with different letters presented significant differences.

## Data Availability

The data presented in this study are available on request from the corresponding authors.
